# Extraction of Acids and Bases from Aqueous Phase to a Pseudoprotic Ionic Liquid

**DOI:** 10.3390/molecules24050894

**Published:** 2019-03-04

**Authors:** Nikolas Patsos, Karin Lewis, Francesco Picchioni, Mark N. Kobrak

**Affiliations:** 1 Department of Chemical Engineering, University of Groningen, Nijenborgh 4, 9747 AG Groningen, The Netherlands; n.patsos@student.rug.nl (N.P.); k.m.lewis@student.rug.nl (K.L.); f.picchioni@rug.nl (F.P.); 2 Department of Chemistry, Brooklyn College of the City University of New York, 2900 Bedford Ave., Brooklyn, NY 10314, USA; 3 Department of Chemistry, Graduate Center of the City University of New York, 365 Fifth Ave., New York, NY 10016, USA

**Keywords:** protic ionic liquids, acid extraction, Hofmeister effect

## Abstract

We report experiments on the extraction of acids and bases from an aqueous phase to a pseudoprotic ionic liquid phase consisting of an equimolar mixture of trihexylamine and octanoic acid. We observed the extraction of a wide range of acids and bases, and investigated the mechanism of extraction in detail. Our results confirmed the observation of the Hofmeister effect in these systems reported in our previous work, where the extent of the extraction of copper salts was significantly influenced by the interactions between extracted inorganic anions and the organic phase. Our results further demonstrated that the organic layer served as a “floating buffer” capable of stabilizing the pH of an acidic or alkaline aqueous phase. The results tie current interest in protic and pseudoprotic ionic liquids to earlier work on the extraction of acids using amine and acid–base couples as extraction agents in an inert organic solvent.

## 1. Introduction

Protic ionic liquids are ionic liquids formed by the mixture of an organic acid and a weak base, with a subsequent proton transfer to create ions [1,2]. These species have been studied extensively in recent years, in a range of applications [3,4,5,6]. A particular question of interest considers when an equimolar mixture should be considered “ionic” (implying that the proton has transferred) rather than “molecular.” While questions remain, a general rule of thumb has emerged that if the difference in pK_a_ between the acid and the conjugate acid formed by the base is greater than 10, then the system should be viewed as ionic [7,8]. This rule is supported by a range of physical measurements.

However, other studies suggest that equimolar mixtures of acids and bases can have odd properties, even when their pK_a_ values and other physical measurements suggest that proton transfer does not occur This can include unusual spectroscopic behavior [9], enhanced conductivity [9], and anomalous dielectric response [10]. Such mixtures have been dubbed “pseudoprotic ionic liquids”.

We have recently conducted studies considering mixtures of trialkylamines and straight-chain organic acids [11,12]. Such systems are similar to mixtures of organic acids and amines that have been studied systematically for a number of decades [13,14,15], though to our knowledge our prior work considers the heaviest examples of such molecules and we are the first to consider their behavior in contact with water or as water-saturated mixtures. The physical properties of these mixtures—both the anhydrous low molecular weight mixtures and the water-saturated higher molecular weight species—show that at certain mole ratios of acid to amine, one observes a maximum in both viscosity and in electrical conductivity. The coincidence of these two phenomena implies the emergence of a hydrogen-bonded network, which both increases the strength of the intermolecular interactions (increasing liquid viscosity) and facilitates conductivity via a rearrangement of covalent and hydrogen bonds analogous to the Grotthus mechanism in water. Recent x-ray scattering experiments have confirmed the existence of nanoscale structures in these liquids, and these structural features are most pronounced where the physical properties suggest the hydrogen bond network is strongest [12].

These nanoscale structures are of interest for both technological and fundamental reasons. We recently explored the utility of mixtures of this type for the extraction of copper salts from aqueous phase, and observed that the extent of the extraction depended significantly on the identity of the anion of the salt. This dependence was consistent with the Hofmeister effect, in which ions stabilize the hydrophobic/hydrophilic interfaces to a degree that depends on their chemical structure [16]. The existence of nanostructures of alternating hydrophobic and hydrophilic character, as might be expected to emerge from amphiphilic molecules of this type, necessarily creates such interfaces. The reduction of the interfacial free energy associated with the presence of the anions creates a driving force for their extraction from the aqueous phase to the (structured) organic phase.

In this work, we extended those studies by considering the extraction of a range of mineral acids and bases from aqueous phase. The working hypothesis, largely confirmed, is that the extraction of acids follows a mechanism similar to that observed in copper salts. The use of acids allows for the exploration of a greater range of anions without concern for compatibility with the metal, and (because all acids employed are strong acids) without the risk of coordination chemistry that could complicate the results. The bases were studied for completeness. Strikingly, in addition to the insight obtained on the extraction, we observed that samples of widely different initial pH converged to the same pH value after extraction. In other words, the presence of the second phase amounted to a “floating buffer” that made the system resistant to changes in pH. The results confirmed the relevance of the Hofmeister series to extraction of the structured organic phase, and also raised the possibility of technological applications for this novel buffer system.

This work ties to another field of study, the liquid–liquid extraction of acids to an organic phase. The most notable examples of this include extractions to solutions containing amines [17,18,19,20] and organic acid–base couples (ABCs) [21,22]. The latter consist of an organic amine and an organic acid dissolved in a low polarity solvent, often a hydrocarbon system such as kerosene. Such systems represent diluted versions of the pure acid/amine system considered here, and the results of those studies indicate a selectivity for mineral acids that is identical to our own and is consistent with the Hofmeister series. We discuss this further in the Results and Discussion.

## 2. Results and Discussion

**Figure 1:**
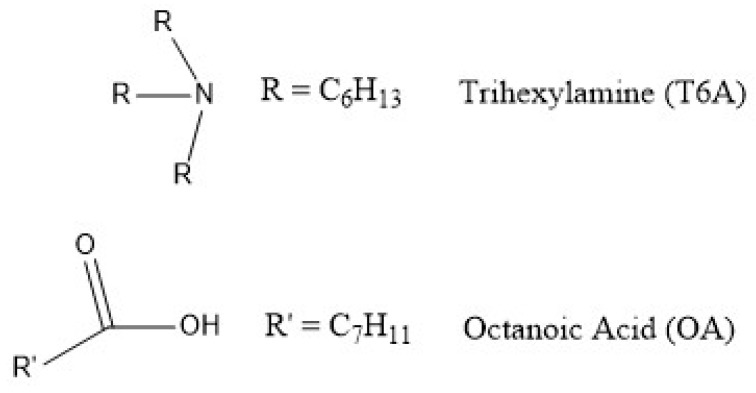
Organic compounds used in this study.

The organic phase used in the present study was an equimolar mixture of trihexylamine and octanoic acid (mixture abbreviated T6A OA), shown in Figure 1.

Two types of experiments have been reported. The first involves biphasic extraction of an acid or a base from an aqueous phase to the organic mixture, with the goal of identifying how the pH of the aqueous phase changes as a function of the initial pH and the identity of the acid or base. The second set of experiments characterizes the contents of the aqueous phase to ascertain the mechanism of extraction.

### 2.1. Extraction of Acids and Bases from Water

Table 1 gives the identity of the acids and the bases used in this study. For each acid, a range of aqueous solutions of differing pH were prepared and extracted. Extractions involved the mixing of 4.00 mL of aqueous phase, with an equal volume of organic phase, followed by centrifugation and separation of the phases by syringe, prior to analysis. See Methodology for details.

**Figure 2:**
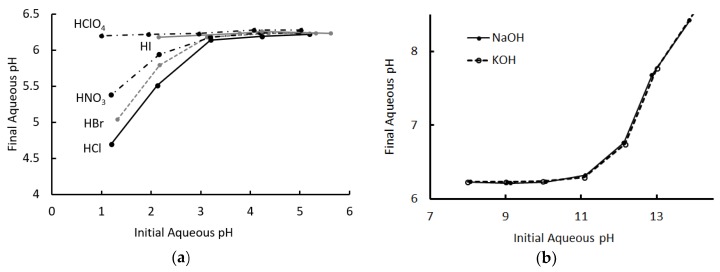
Final pH of solution after extraction, as a function of the initial pH. (**a**) Acid extraction. Data for the extraction of hydroiodic acid for pH < 2 was not reported, as the organic phase showed evidence of decomposition in this experiment. (**b**) Base extraction. Aqueous and organic phases were of equal volumes in these experiments.

Figure 2 shows the initial and final pH values for all experiments, for each acid and base. Looking first at the acids, the pH converged, consistently, to ~6.2 in each case where the initial pH was between 6 and 3. The final pH was independent of the identity of the acid over this range, in a manner that was remarkably like that of a conventional monophasic buffered system.

For extractions with an initial pH less than 3, the final pH began to diverge from 6.2 to a degree that depended on the identity of the acid. Even for HCl, the least effectively buffered system, there was still a significant neutralization of the aqueous phase, but an order was clearly visible. The results of extraction for hydroiodic acid for pH < 2 are not reported, as there was evidence of degradation of the organic phase under these circumstances.

To make this trend more transparent, we have re-expressed some of the data from Figure 2a in Table 2. The results for experiments of pH less than three have been reported, along with a calculated distribution coefficient D_acid_, representing

D_acid_ = [H^+^(org)]/[H^+^(aq)] (1)

The chemical identity of the hydrogen ion in the organic phase was unknown, though the most likely possibility was that it existed primarily bound to a trihexylammonium cation. The concentration of hydrogen ions in the organic phase was inferred from the aqueous phase concentration and the initial volumes of the organic phases, and so did not account for small changes in volume as the organic phase took up water during extraction. Nevertheless, the estimate was reasonable and allowed a comparison of the extent of extraction of each acid. It is interesting to note that the pattern of most- to least-effectively buffered matched the Hofmeister series [16], a point we return to in Section 2.3.

A similar pattern emerged for the bases, which also converged to a pH of ~6.2 for samples with initial pH values between 8 and 11, at which point they began to diverge. Unlike the acids, the two bases used appeared to behave identically and the cation did not appear to influence the outcome.

### 2.2. Mechanism of Extraction

In order to study the mechanism of extraction in greater detail, a set of extraction experiments were conducted at a high concentration, with organic phases consisting of pure octanoic acid and pure trihexylamine used as controls. Experiments thus consisted of an organic phase (pure amine, pure organic acid, or a 1:1 molar mixture of each) used to extract acid or base from an equal volume of water. For these experiments, both the pH of the aqueous phase and the concentration of the remaining counter-ion (chloride for HCl, sodium for NaOH) were measured. The results are reported in Table 3 and Table 4, and the details of the method are described in Section 3.2.

The results for the extraction from basic solution are shown in Table 3. Looking first at the extraction to trihexylamine, we note that the final aqueous concentration of sodium was higher than the initial value. This likely reflected contamination in the trihexylamine prior to use, as the manufacturer certified only 96% purity; it might also be the result of sodium leeching from the glass storage vessel in the presence of a strongly basic solution. Regardless, it was clear from the high concentration of both sodium and hydroxide ions in the aqueous phase that there was no extraction of sodium hydroxide by trihexylamine.

In contrast, the concentration of sodium dropped dramatically in the presence of octanoic acid, and the hydroxide nearly vanished from the aqueous phase. In a previous study [12] we have found that pure octanoic acid takes up a substantial amount of water, and that the water-saturated octanoic acid displays nanostructural features with lengthscales on the order of the chain length of the acid. This implies the existence of domains of significant size possessing an aqueous character, which could provide an environment for the solvation of sodium ions within the octanoic acid. The limited extraction of Na^+^ and the near-complete neutralization of the aqueous phase suggested that two mechanisms were in operation, to varying degrees:

NaOH(aq) + HOA(org) ⇋ NaOA(aq) + H_2_O(l) (2a)

NaOH(aq) ⇋ NaOH(org)(2b)

The term (org) denotes dissolution in the organic phase. The solubility of sodium octanoate in the aqueous phase was quite high (critical micelle concentration ~0.4M [23]), making the former mechanism plausible. The discrepancy between the extent of neutralization and the sodium extraction could be explained by the fact that while both mechanisms neutralized the base, only the second one reduces the [Na^+^(aq)]. The processes described here are similar to those observed in our earlier studies of copper extraction [11], where it was found that the extent of each mechanism depended on the initial concentration of the salt to be extracted. Similar competing mechanisms were observed in extractions of ionic liquids [24,25].

The same mechanisms can be used to understand the results for the mixture T6A OA. In the latter case, only a small amount of sodium was extracted, and the dominant mechanism is likely to be that described in Equation (2a). This was consistent with the observation that NaOH and KOH were neutralized to identical degrees by T6A OA, as the alkali metal remained in the aqueous phase and, therefore, amounted to a spectator ion in the basic extraction process.

Turning now to the extraction of acid reported in Table 4, it seems clear that extraction to trihexylamine proceeds primarily by the reaction

HCl(aq) + T6A(org) ⇋ HT6ACl(org)(3)

In other words, trihexylammonium chloride becomes dissolved in the organic phase. As noted in the Introduction, the ability of amines to extract acids is well-known. The small discrepancy between the final aqueous [Cl^−^(aq)] and the [H^+^(aq)] is likely explained by the dissolution of a small quantity of the (largely aqueous-insoluble) T6A in the aqueous phase. This would neutralize the hydrogen ion without affecting the concentration of chloride. As in the case of the octanoic acid neutralization of the base, two mechanisms are in play, albeit to a very limited degree owing to the low solubility of T6A in the aqueous phase.

The results for octanoic acid revealed that essentially no acid is transferred to the organic phase. While not surprising, it is worth noting that the high water content of the octanoic acid and the existence of a nanoscale structure made it possible to conceive of mechanisms for the dissolution of the acid in a nanoscale aqueous phase. The experiment ruled out this possibility.

Turning now to the mixture, we note three possible mechanisms for the neutralization of the aqueous solution by the T6A OA given in Equation (4a–c): Anion exchange, neutral extraction, and cation exchange. The anion exchange mechanism neutralizes the aqueous phase by the transfer of octanoate ions to the aqueous phase, and their subsequent reaction as a weak base. However, octanoic acid was soluble in water only to approximately 5 mM [26], which limited the extent of neutralization by this mechanism (neutralization followed by a transfer of the octanoic acid back to the organic phase would lead to the same net equation as the neutral extraction).

Neutral extraction appeared to be plausible, with the H^+^ in the organic phase likely associating with the trihexylamine, and the chloride associated with the aqueous nanodomains within T6A OA. The mechanism required a 1:1 ratio of extracted H^+^ and Cl^−^, implying that if it were the sole mechanism of operation, there would be an equimolar amount of both ions in the aqueous phase. Thus, while neutral extraction might account for a significant fraction of the total extraction, there must be an additional mechanism at work to account for the fact that at equilibrium, [H^+^ (aq)] < [Cl^−^ (aq)].

This can be explained by the cation exchange mechanism, which was made possible by the presence of Na^+^ (org) in the amine, as noted above. While the low fraction of aqueous chloride ion remaining in the aqueous solution implied that the neutral extraction mechanism was dominant, the discrepancy between hydrogen and chloride ion concentrations made it clear that the cation exchange mechanism was also significant.

Cl^−^ (aq) + OA^−^ (org) ⇋ OA^−^ (aq) +Cl^−^ (org) (4a)

HCl (aq) ⇋ HCl (org)(4b)

Na^+^ (org) + H^+^ (aq) ⇋ Na^+^ (aq) + H^+^ (org) (4c)

### 2.3. Role of the Hofmeister Effect

Figure 2a indicates that the extent of acid extraction from the aqueous phase, at a low pH, strongly depended on the identity of the anion. This was consistent with the observation of the dominance of the neutral extraction mechanism in the acid extraction, as the acid anion was transferred to the organic phase and its solvation energetics should therefore have influenced the extent of the reaction. Similarly, the dominant mechanisms for neutralization of the base by the T6A OA phase did not involve transfer of the metal cation. This explained why the extent of extraction appeared to be independent of the identity of the cation, as evidenced by Figure 2b.

The dependence of the extent of acid extraction on the identity of the anion can be understood with reference to the Hofmeister effect [16], in which certain ions are known to stabilize hydrophobic/hydrophilic interfaces to varying degrees. The Hofmeister effect has been observed in many different interfacial phenomena, including micellization [27], salting in/out of proteins [28], and chromatography [29]. Different ions stabilize such interfaces to different extents, but the relative degree of stabilization offered by a given ion is very consistent, even in widely different contexts. In other words, one may rank ions based on their ability to stabilize an interface, and this ranking is known as the Hofmeister series.

Our previous work on the T6A OA system [12] demonstrated that the mixture is nanostructured, with features on a lengthscale of ~1.8 nm. Physical characterizations in the same work implied the existence of a hydrogen-bonding network, and given the amphiphilic nature of the components of the mixture these results made clear that the structures in question possessed domains of alternating hydrophobic and hydrophilic character. This, in turn, implied the existence of an interface between these domains.

The extent of acid extraction from the low pH solution shown in Figure 2a exactly matched that of the Hofmeister series for the five ions in question, with chloride stabilizing the interface least effectively and perchlorate stabilizing it to the greatest degree. The Hofmeister effect was important in these experiments due to the presence of the hydrophobic/hydrophilic interface in the organic phase. The transfer of anions to the organic phase stabilized this interface, a phenomenon that provided a free energy driving force for the extraction of the acid. The magnitude of the driving force depended on the extent to which the interface was stabilized, and thus followed the Hofmeister series. The effect was only detectable when the organic phase had begun to saturate with acid (i.e., at low initial pH), but the pattern was consistent.

The Hofmeister effect is also known for cations and might have been expected to be relevant for the neutralization of bases. However, as discussed in Section 2.3, the metal ions were not transferred to the organic phase for T6A OA. Thus, they did not interact with the nanostructures in the organic phase and so the Hofmeister effect was irrelevant.

### 2.4. Comparison to Other Acid Extraction Systems

As noted in the Introduction, this work was similar to work on extractions to solutions containing amines [17,18,19,20] and organic acid–base couples (ABCs) [21,22]. These studies analyzed the extent of extraction, using different schemes from that reported here (e.g., assigning an effective pKa to the organic phase), so it was unclear whether these systems acted as buffers or how their neutralization capacity should be compared. However, these studies did consider the selectivity of the extraction for different mineral acids, supporting a comparison with the Hofmeister effect observed here.

Looking first at the extraction of acids from amines dissolved in hydrocarbons, we noted that Bertocci and Rolandi [18] considered the extractions of a series of mineral acids with trioctylamine in xylene. The acids were extracted in the order HNO_3_ > HCl > H_2_SO_4_; the authors also reported results for HF, but noted idiosyncrasies that made a comparison moot. The order of the strong acids matched the Hofmeister series, and was consistent with the results discussed here. It is unclear whether the underlying cause of this ordering is the same, however. The authors did not consider the possible role of aggregation or the development of nanophases, though they did observe very specific ratios between amine, acid, and water dissolved in the organic phase which could correlate with the emergence of well-defined aggregate structures. In a similar study of tertiary amines in a chloroform solution, Moore [20] made similar observations. The author reported a similar ordering with respect to mineral acids, with the order of greatest extraction generally following the order expected by the Hofmeister series: HNO_3_ > HCl > H_2_SO_4_ > H_3_PO_4_. There was some ambiguity, however, as in cases where the difference in the extent of extraction was very small (<2% as measured in experiments) the Hofmeister series was violated in some cases.

Intriguingly, in a similar study, Grinstead and Davis [17] observed a concentration-dependence on the extent of extraction that they attributed to aggregation of the amine. The aggregation effects were most pronounced for the tertiary amines, and the authors suggested a substantial trialkylammonium/chloride ion pairing and the possible inclusion of water in the aggregates as important effects. While the authors considered only hydrochloric acid in their study, the evidence of well-defined aggregate structures supports our proposal that the formation of nanostructures might be an important factor in acid extraction phenomena.

Turning to ABC extractions, Eyal and Baniel [21] considered various amines in mixtures with a range of organic acids diluted by a series of organic solvents. Their results demonstrated that the extraction system was selective for HNO_3_ over HCl, and H_2_SO_4_ and HCl were preferred over H_3_PO_4_. Other work observed a strong selectivity of HCl over H_3_PO_4_ [22]. With the exception of the extent of extraction for H_3_PO_4_ relative to H_2_SO_4_ (which might relate to their polyprotic character), these results are consistent with the Hofmeister series and the results reported here.

The significance of these results is unclear for both the amine extractants and for the ABC systems. There is insufficient evidence to infer that the same effects are at work in those systems and in the neat amine/acid mixtures studied here. Nevertheless, the possibility of the emergence of hydrogen-bond networks associated with amphiphilic aggregates, and the possibility that those aggregates could represent nanoscale structures, cannot be ruled out. This might be a fruitful line of inquiry for future work.

## 3. Materials and Methods

### 3.1. Methodology for Extraction Experiments

Octanoic acid (Acros Organics, Pittsburgh, PA, USA) and trihexylamine (Sigma Aldrich, St. Louis, MO, USA) were used as received for the preparation of the T6A OA organic mixture. Hydrochloric acid (37%, Merck, Kenilworth, NJ, USA), nitric acid (65%, Merck), bromic acid (47%, Merck), hydroioidic acid (55%, BDH Chemicals, Radnor, PA, USA), and perchloric acid (70%, Acros Organics) were diluted with Milli-Q water (deionized with Simplicity Millipore deionizer, Millipore, Burlington, MA, USA) to the pH values at which they were used. Sodium hydroxide (>99%, Merck) and potassium hydroxide solution (87.5%, J. T. Baker, Radnor, PA, USA) were similarly diluted.

Trihexylamine and octanoic acid were mixed in 1:1 mole ratio, based on weighings with an analytical balance. After initial mixing, samples were stirred 20+ hours, using a magnetic stirrer.

For the extraction experiments, 4.00 mL of the organic phase were mixed with an equal volume of the aqueous phase and shaken, overnight on a Beun De-Ronde wrist-shaker. Phases were then isolated using a Medilite centrifuge, and the resulting phases were separated by a syringe.

pH measurements of the aqueous phase were conducted with a Mettler-Toledo Seven2Go apparatus (Columbus, OH, USA) and a a Mettler-Toledo InLab Expert electrode. The pH meter was calibrated, prior to each round of measurements, using Certipur buffer standards (Merck).

### 3.2. Methodology for Mechanism Experiments

The identities of the reagents and the procedure for preparation of T6A OA, followed the same protocols as used in Section 3.1. A total of 1.00M hydrochloric acid (Fisher, Titripur, Pittsburgh, PA, USA) was used as received, and 1.00M sodium hydroxide was prepared from solid NaOH (Sigma-Aldrich), by dilution in Milli-Q water. Five milliliters of the aqueous phase (acid or base) were placed in contact with an equal volume of the organic phase of either trihexylamine, octanoic acid, or T6A OA. The solutions were stirred together for 1 h, and allowed to sit overnight, to separate the phases. Aqueous phase was drawn off by a syringe.

Ion chromatography measurements were performed on a Dionex LC25 unit (Sunnyvale, CA, USA), with an AS14 5 μm column for the anions and a CS12A 5 μm column for the cations. Where dilutions were necessary, samples were diluted using Ultra Purified deionized water.

## 4. Conclusions

This work considered both fundamental and applied questions. From the standpoint of fundamental research, the work validated our prior demonstration of the relevance of the Hofmeister effect to extraction [11] by both widening the range of ions considered and using a non-metal cation to avoid potential complications arising from coordination chemistry. The confirmation that the Hofmeister effect is relevant to extraction phenomena involving nanostructured fluids will both aid in the interpretation of experimental results in the area and potentially create new strategies to drive extraction.

However, the discovery that liquids of the type reported here act as “floating buffers” raises its own intriguing possibilities for applications. Conventional buffers, based on the mixing of a weak acid and its conjugate base, have a buffer capacity determined by the ionic strength of the aqueous phase. The system derived here does not suffer from the same restriction. Though we have not explored this aspect, it seems likely that the buffer capacity can be increased by increasing the volume of the organic phase relative to the aqueous phase, a process that does not involve altering the ionic strength of the aqueous phase. While there might be other complications to using this system, such as the contamination of the aqueous phase by trace organics, these may be acceptable in cases where the ionic strength is a hindrance. Consider, for example, that enzyme catalysis is often active only over a limited pH range and might be sensitive to ionic strength. The scheme presented here sidesteps the latter issue and might, therefore, be suitable in applications that are served poorly by conventional buffer solutions.

## Figures and Tables

**Table 1:** Acids and bases extracted from the aqueous phase.

Acid	Base
HBr	KOH
HCl	NaOH
HClO_4_	
HI	
HNO_3_	

**Table 2:** Distribution coefficients as a function of initial pH and identity of the acid.

Acid	Initial Aqueous pH	Final Aqueous pH	D_acid_
HClO_4_	1.0	6.2	1.6 × 10^5^
HNO_3_	1.2	5.4	1.5 × 10^4^
HBr	1.3	5.0	5.3 × 10^3^
HCl	1.2	4.7	3.2 × 10^3^
HClO_4_	2.0	6.2	1.9 × 10^4^
HI	2.2	6.2	1.1 × 10^4^
HNO_3_	2.2	5.9	6.0 × 10^3^
HBr	2.2	5.8	4.2 × 10^3^
HCl	2.1	5.5	2.4 × 10^3^

**Table 3:** Aqueous phase composition after extraction from basic solution. Initial aqueous phase: 1.00M NaOH.

Organic Phase	Final Aqueous [Na^+^(aq)] (M)	[OH^−^(aq)] (M)
Trihexylamine	1.18 +/− 0.01	1.1
Octanoic Acid	0.0893 +/− 0.001	5.0 × 10^−8^
T6A OA	0.76 +/− 0.01	2.5 × 10^−6^

**Table 4:** Aqueous phase composition after extraction from acidic solution. Initial aqueous phase: 1.00M HCl.

Organic Phase	Final Aqueous [Cl^−^(aq)] (M)	[H^+^(aq)] (M)
Trihexylamine	1.09 × 10^−4^ +/− 1 × 10^−6^	4.9 × 10^−5^
Octanoic Acid	1.0 +/− 0.1	1.0
T6A OA	0.056 +/− 0.001	6.5 × 10^−5^
